# Surface tension measurements reveal charge-driven surfactant depletion in microdroplets approaching the Rayleigh limit

**DOI:** 10.1039/d6sc03152h

**Published:** 2026-06-11

**Authors:** Firoz Ahmed, Michael I. Jacobs

**Affiliations:** a Department of Chemistry and Biochemistry, Texas State University San Marcos TX 78666 USA mijacobs@txstate.edu

## Abstract

The net charge on droplets is predicted to play a crucial role in accelerating chemical reactions in microdroplets by either altering reaction energetics or changing surface compositions. However, there are few experimental studies that have explored how the net charge of microdroplets alters their physicochemical properties. Here, we present a new technique to investigate how net charges on levitated microdroplets affect their surface tensions and the partitioning of surfactants to the air–water interface. The technique is validated by measuring how the resonant surface oscillation frequency (*ω*_obs_) of microdroplets changes with increasing net charge. For simple one- and two-component microdroplets, *ω*_obs_ predictably decreases with charge. This enables the determination of surface tension of microdroplets (6–20 µm radius) with net charges approaching the Rayleigh limit (*Q*_R_), which represents the maximum net charge a droplet can stably carry before coulombic fission. To demonstrate how this new technique can be used to measure charge-driven changes in surface composition, we measured the dependence of surface tension on charge for microdroplets containing cetrimonium bromide (CTAB). For ∼8 µm-radius microdroplets containing 0.5 mM CTAB, the surface tension was observed to increase from ∼50 to ∼65 mN m^−1^ as net positive charge on droplets increased from low charge to *Q*_R_. A similarly-sized change in surface tension was not observed in negatively charged droplets nor droplets with CTAB concentrations larger than the critical micelle concentration. These initial results demonstrate that the net charge on droplets can alter the equilibrium partitioning of charged surfactants to the air–water interface. Ultimately, the new approach to measure the surface tensions of highly-charged microdroplets enables investigations into how charge alters interfacial concentrations of molecules to promote microdroplet chemistry.

## Abstract 2

New surface tension measurements of highly-charged microdroplets demonstrate that surface partitioning behavior of charged surfactants is strongly influenced by net microdroplet charge close to the Rayleigh limit.

## Graphical abstract

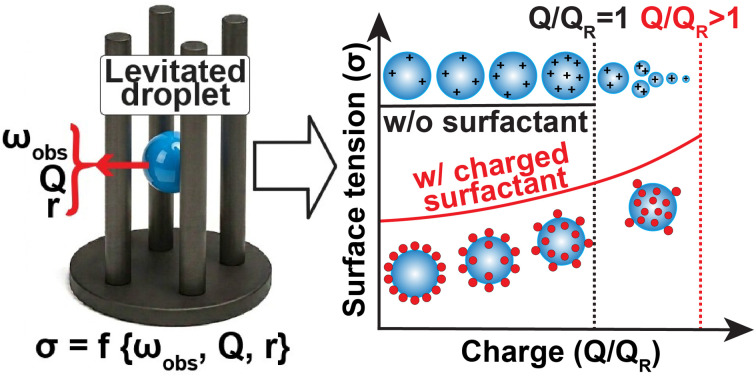

## 1. Introduction

Charge is believed to play a fundamental and transformative role in microdroplet chemistry, driving chemical reactivity in microdroplets along pathways that differ significantly from those observed in bulk-phase systems. Numerous chemical reactions have been observed to occur up to six orders of magnitude faster in charged microdroplets than in macroscale solutions.^1–4^ It is currently thought that chemistry is promoted in microdroplets due to their the large, exposed surface areas.^5,6^ Charges at the air–water interface can alter the properties of the surface to promote chemistry.^2^ For example, excess H_3_O^+^ and OH^−^ charges at the air–water interface can behave as ‘superacids’ or ‘superbases’ to promote acid/base chemistry in microdroplets.^2,7–11^ In addition, coulombic fission of charged droplets generates small daughter droplets with pH values strongly influenced by the unique acid–base character of the air–water interface.^12^ Net charges in droplets are also thought to contribute to the formation of strong electric fields at droplet surfaces which can alter electron transfer thermodynamics to promote redox chemistry.^1,3,13–18^ Finally, net charges in microdroplets can enhance molecular concentrations at the air–water interface through the creation of an electric double layer at the surface and enhanced molecular transport of charged species to the surface.^19–23^ Ultimately, depending on the exact physicochemical mechanism driving accelerated chemistry, the unique chemistry observed in charged microdroplets could have broad applications ranging from explaining atmospheric phenomena such as thunderstorm electrification^24^ to spontaneous hydrogen peroxide formation^25,26^ to nanomaterial synthesis^27,28^ to rapid chemical screening for drug design and formulation.^3^

Several techniques are widely available for generating charged microdroplets, including electrospray ionization (ESI),^29,30^ ultrasonic atomization,^31^ and gas nebulization.^32^ Among these, ESI has become the most popular technique to generate charged microdroplets. ESI involves applying a high voltage to a liquid at a fine capillary tip, forming a Taylor cone that emits a charged jet that breaks into charged microdroplets.^29^ Ultrasonic atomization uses high-frequency acoustic waves to eject droplets, where cavitation-driven ion segregation imparts a net charge to the droplet.^33,34^ Alternatively, gas nebulization uses high-velocity gas to shear liquid into droplets.^35^ These droplets undergo triboelectric charging and ion evaporation, processes that can be intensified by using ionized carrier gases or applying an external electric field near the nozzle.^36,37^ In addition to direct generation of charged microdroplets, highly charged droplets may also form when neutral droplets undergo fragmentation or interact with surfaces.^38–40^ Ultimately, droplets from each of these sources not only have large exposed surface areas to promote interfacial chemistry and facilitate transport^41^ but also have large net charges that can influence reaction thermodynamics or alter surface compositions.

The magnitude of charge not only influences reaction rates and pathways but also determines the stability of droplets. The maximum charge a single microdroplet can hold before becoming unstable was first described by Lord Rayleigh in 1882.^42^ Rayleigh predicted that charged droplets should become unstable when the “fissility parameter” *X* = *Q*^2^/64π^2^*σε*_0_*r*^3^ (which represents the ratio of electrostatic energy of the droplet to twice its surface energy) exceeds 1. Here, *Q*, *ε*_0_, *σ*, and *r* represent the net charge on a droplet, the permittivity of free space, surface tension and droplet radius, respectively. When the repulsive force from the net droplet charge exceeds the cohesive force of surface tension, the droplet undergoes coulombic fission, fragmenting a mother droplet into one or more progeny droplets.^43–47^ A fissility parameter *X* = 1 represents the onset of charge-induced droplet instability and is used to determine the critical charge threshold known as the Rayleigh limit, *Q*_R_:1
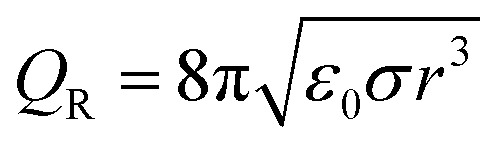


The Rayleigh limit *Q*_R_ represents the maximum net charge a droplet can stably carry and can be used to describe the fissility parameter *X* = *Q*^2^/*Q*_R_^2^ of a charged droplet. Many studies have directly measured the Rayleigh limit in levitated charged microdroplets and found droplet fission typically occurs at or slightly below the theoretical Rayleigh limit.^14,43,45–51^

A central limitation in harnessing charged microdroplet reactivity is their inherent electrostatic instability; microdroplet reactivity is believed to increase as the net charge on droplets approaches the Rayleigh limit,^1^ but the finite capacity of surface tension forces limits how much charge can be added to microdroplets. Increasing charge at droplet surfaces is predicted to re-organize the spatial distribution of charged molecules within droplets, potentially altering the chemical composition of the air–water interface and reactivity of microdroplets.^19,21,22^ Indeed, previous studies have shown that increasing the net charge on droplets can induce nucleation of solutes, suggesting net charges can drive changes in surface concentrations or orientations of molecules.^52–54^ Changing surface composition could alter droplet surface tension, which would affect how much charge a droplet could stably carry. Thus, experimental techniques capable of measuring how droplet surface compositions change with increasing net charge are crucially needed to understand the mechanisms driving accelerated chemistry in highly charged microdroplets.

In this work, we describe a technique to measure the surface tensions of levitated microdroplets with charges approaching *Q*_R_. Using the formalism described by Rayleigh, we relate measured microdroplet sizes, surface oscillation frequencies and net charges. First, to validate the ability of this technique to measure the surface tensions of highly charged microdroplets, we explore the effect of charge on surface oscillation frequency for levitated microdroplets with three chemically distinct compositions: (1) aqueous droplets with high ionic strength (3 m NaCl), (2) aqueous droplets with low ionic strength (30% (w/w) glycerol–water), and (3) non-aqueous, organic liquid droplets with a low dielectric strength (hexadecane). For these chemical systems, microdroplet surface tension remains constant with increasing charge, validating the technique. To demonstrate how this new technique can be applied to microdroplet chemistry, we then measure how the surface tensions and, by extension, surface compositions of surfactant-laden microdroplets change with increasing net charge.

## 2. Experimental section

### 2.1 Sample preparation

Aqueous stock solutions of NaCl (Fisher), glycerol (Fisher) and cetrimonium bromide (CTAB, Tokyo Chemical Industry) were prepared using ultrapure 18.2 MΩ cm water. Hexadecane stock solution was prepared by mixing ethanol (Fisher), formic acid (Fisher) and hexadecane (Fisher) in a volume ratio of 90 : 5:5 (v/v%), respectively.

### 2.2 Macroscopic surface tension measurements

Equilibrium surface tensions of macroscopic solutions were measured with either the Wilhelmy plate method (Kruss K20) or the pendant drop method (OpenDrop^55,56^) operating at room temperature. For CTAB solutions, the surface tension was measured as a function of concentration and a Frumkin isotherm was used to relate bulk solution concentrations to surface concentrations, as previously described.^57^

### 2.3 Microscopic surface tension measurements

Numerous surface tension measurements of microdroplets have been used to inform on either the dynamic^58^ or equilibrium^57,59–65^ surface composition of microdroplets.^66^ In general, these techniques measure the surface oscillation frequencies of levitated microdroplets and relate them to surface tensions using formalism that was first described by Rayleigh.^42^ Using this framework, the observed surface oscillation frequency (*ω*_obs_) of a charged droplet is composed of two components: the natural oscillation frequency (*ω*_0_) arising from surface tension forces and the electrostatic repulsion contribution (*ω*_C_) arising from the droplet's charge:

2 *ω*_obs_^2^ = *ω*_0_^2^ + *ω*_C_^2^

For the *l*-th oscillation mode, *ω*_0_ and *ω*_C_ are described by:3
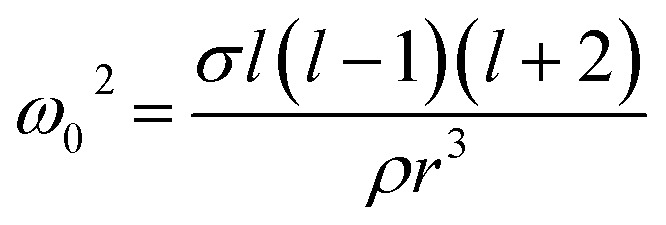
4
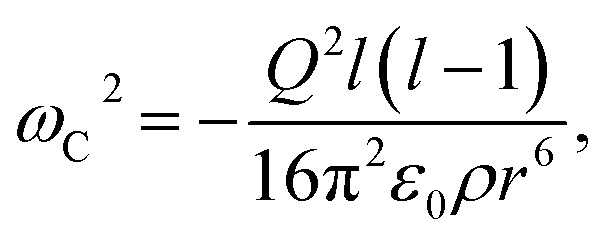
where *ρ* is the density of the microdroplet and all other variables have been previously defined. Previous studies exploring the surface tensions of levitated microdroplets have used either uncharged or marginally charged droplets (<10% of *Q*_R_) and have neglected the *ω*_C_^2^ term from analysis. However, as the net charge on microdroplets increases, it is increasingly important to consider the electrostatic repulsive contribution to accurately determine surface tension (and by extension surface compositions) from droplet surface oscillation frequency.

### 2.4 Electrodynamic microdroplet levitation

**Fig. 1:**
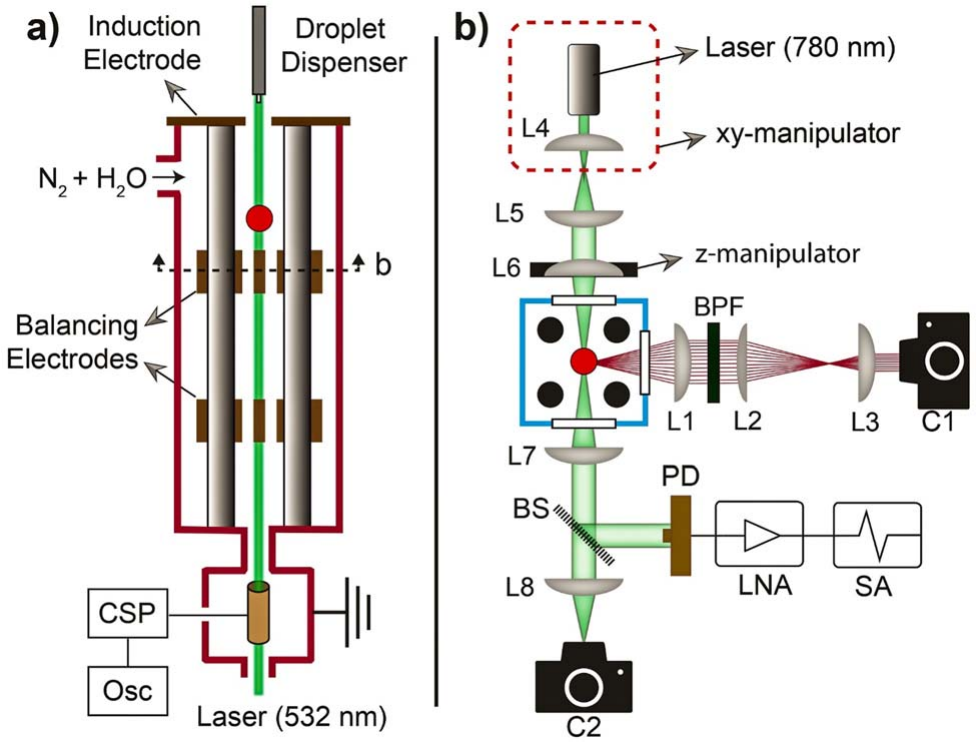
(a) Experimental setup of the QET used in this work. A droplet (red dot) was levitated for QELS and sizing measurements. The CSP was used to detect the charge of droplets as they were ejected from the trap, with signal acquisition performed *via* an oscilloscope (Osc). (b) Optical setup of the QET. Mie scattering signal was imaged using Camera 1 (C1). A 780 nm laser was focused on the droplet and scattered signal intensity was detected using a photodiode (PD). After amplifying the signal with a low noise amplifier (LNA), QELS spectra were collected with a spectrum analyzer (SA). Camera 2 (C2) was used to position the droplet for QELS measurements.

A quadrupole electrodynamic trap (QET) is used here to measure the size, charge, and surface oscillation frequency of levitated microdroplets. The QET used in this work is similar to those that have been previously described in detail,^67–69^ and a schematic is shown in Fig. 1a. The QET is housed in an environmental chamber, and the relative humidity (RH) was set to the water activity of the different mixtures −90% RH (*a*_w_ ∼0.9) for aqueous systems and 0% RH (*a*_w_ = 0) for hexadecane. Charges are induced on microdroplets by operating a piezo dispenser (30 or 50 µm orifice, Microfab Technologies) close to an induction electrode (±200–2000 V). Charged droplets are trapped axially within the QET by applying an AC potential (300–800 V_ac_, 200–2000 Hz) to quadrupole rods, and droplets are levitated by applying a DC potential to balancing blade electrodes that protrude through the quadrupole electrodes (±20–500 V). A 532 nm laser is directed along the axis of the trap, and the size of the trapped droplet is determined from the angular spacing of Mie scattering fringes using the geometrical optics approximation.^70,71^ A 780 nm laser (100 mW, Civil Laser) is focused on the trapped droplet and quasi-elastic light scattering (QELS) spectra (Fig. 1b) are obtained to determine the resonant surface oscillation frequency of the trapped droplet, as previously described.^57,72–74^ After a QELS measurement, the balancing electrode voltage is removed, and the droplet falls from the trap, passing through a floating charge detection electrode. The charge on the microdroplet is directly determined by measuring the charge induced on the electrode using a home-built charge sensitive pre-amplifier (CSP).^75^

To obtain droplets of different sizes from a single droplet dispenser, mixtures were diluted with water or ethanol (containing 5% formic acid) prior to droplet generation. Excess water or ethanol/formic acid completely evaporated prior to QELS measurements and droplets equilibrated to the water activity of the trap. In this way, droplets with radii varying by roughly a factor of ∼3 could be generated from the same droplet dispenser. In general, droplet sizes ranged from ∼6 to 20 µm radius and net charges (both positive and negative) ranged from ∼10 s to 100 s of fC.

## 3. Results and discussion

### 3.1 Surface oscillation frequency and fissility parameter measurements

**Fig. 2:**
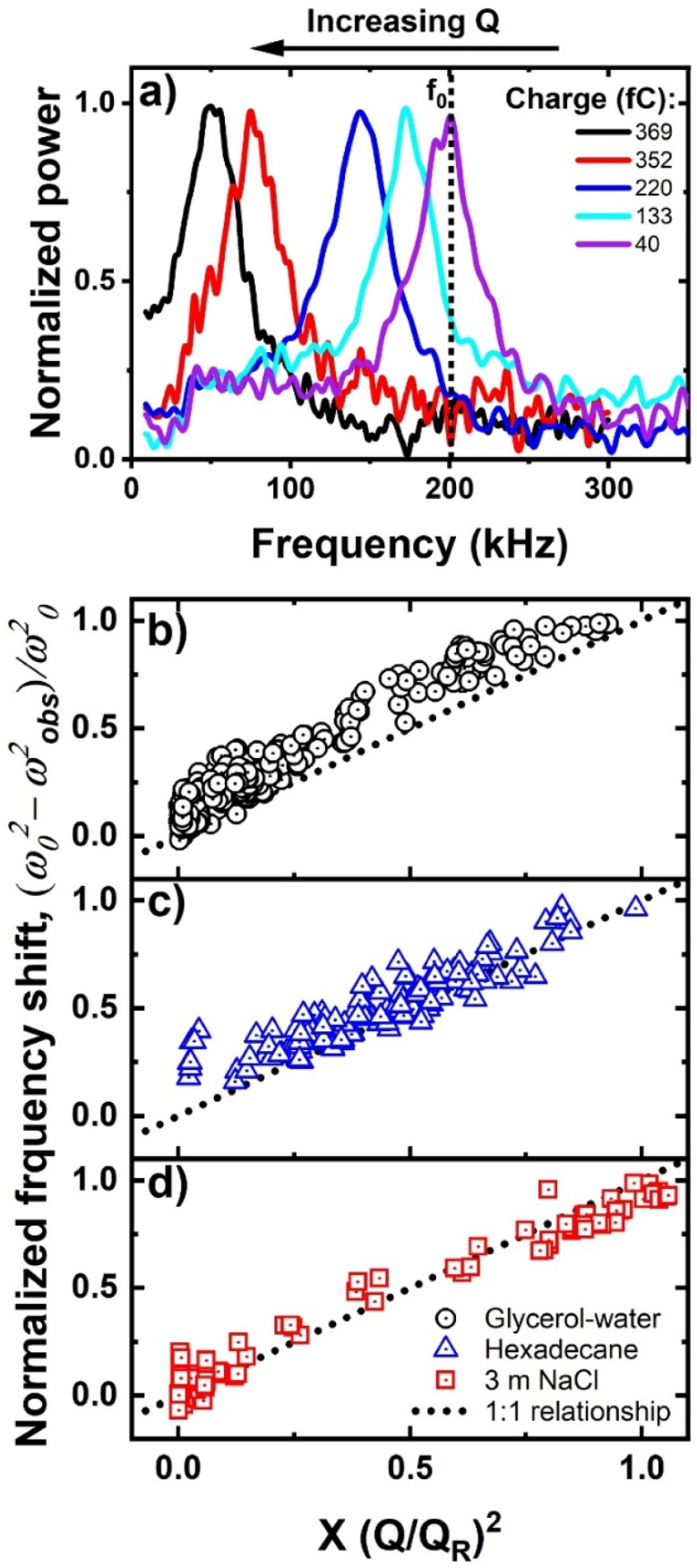
(a) Example QELS spectra for differently charged 7.0 ± 0.2-micron-radius microdroplets containing 3 m NaCl. The vertical dashed line represents the calculated surface oscillation frequency for uncharged droplets. The normalized electrostatic repulsion frequency *ω*_C,rel_^2^ as a function of droplet fissility for microdroplets containing 30% glycerol–water (b), hexadecane (c) and 3 m NaCl (d).

To relate changes in surface tension of highly-charged microdroplets to changing concentrations of molecules at the air–water interface, we first needed to validate the formalism described by Rayleigh that relates surface tension to microdroplet size, charge, and surface oscillation frequency. Previously, surface oscillation frequencies of millimeter-sized, diamagnetically levitated water droplets were measured as function of charge and the formalism predicted by Rayleigh was validated for the first seven oscillation modes using aqueous droplets with charges up to 30% of *Q*_R_ (*i.e.*, *X* < 0.1).^76^ To demonstrate the formalism holds for smaller charged droplets with larger fissilities and different compositions, we measured the size, surface oscillation frequency, and charge of simple one- or two-component microdroplets with net charges ranging from <5% of *Q*_R_ to just below *Q*_R_. As predicted by Rayleigh and previously observed for millimeter-sized charged droplets,^42,76^ the observed surface oscillation frequency *f* = *ω*_obs_/2π of charged microdroplets decreases as the net charge on microdroplets increases (Fig. 2a). As droplet charge increases, coulombic repulsion forces counteract the cohesive influence of surface tension, which reduces the net restoring force required for capillary oscillations. As a result, for a fixed droplet size, the magnitude of *ω*_C_^2^ increases with *Q*^2^ which is readily observed as a shift to lower QELS frequency *f*.

While *ω*_C_^2^ cannot be directly measured, it can be calculated from the difference between *ω*_0_^2^ (calculated from eqn (3) using measured droplet size and macroscale surface tension, Table 1) and observed oscillation frequency *ω*_obs_^2^. To account for variations in droplet size this difference can be normalized by *ω*_0_^2^ to yield a relative electrostatic repulsion frequency, *ω*_C,rel_^2^:5
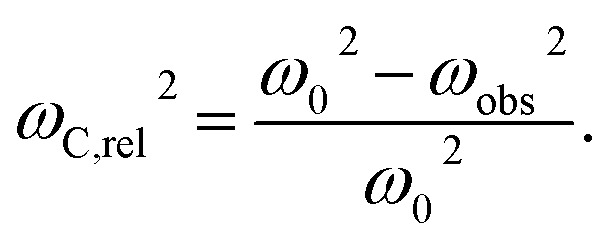
when calculated in this way, *ω*_C,rel_^2^ reflects the relative importance of the electrostatic repulsion contribution to surface oscillation frequency and should vary between 0 (uncharged droplet) and 1 (charged droplet at the Rayleigh limit). Using the formalism presented by Rayleigh, eqn (2) and (3) can be used to show *ω*_C,rel_^2^ = *X* for surface oscillations of mode *l* = 2. To validate this relationship, experimental *ω*_C,rel_^2^ values were plotted as a function of calculated droplet fissility parameter *X* for the three droplet compositions studied here (Fig. 2b–d). Regardless of droplet composition (low ionic strength aqueous, high ionic strength aqueous, and small dielectric non-aqueous) or charge polarity (Fig. S1), the droplets studied here showed a consistent 1 : 1 relationship between *ω*_C,rel_^2^ and *X* = *Q*^2^/*Q*_R_^2^, supporting the predictions made by Rayleigh and previously observed for droplets with small values of *X*.^76^

**Table 1:** Comparison of physical properties for the three droplet compositions studied here

Droplet compositions	Macroscale surface tension (mN m^−1^)	Density (kg m^−3^)	Dynamic viscosity (mPa s)	Measured droplet size (µm)	Observed *Q*_R_	Ohnesorge number (Oh)
30% glycerol–water	67.9 ± 0.9	1070 (ref. 80)	2.50 (ref. 81)	6–20	0.96	0.12–0.07
Hexadecane	26 ± 1	770	2.95 (ref. 82)	20	0.99	0.15
3 m NaCl	76.4 ± 0.7	1100 (ref. 83)	1.34 (ref. 84)	7–20	1.05	0.06–0.03

Importantly, eqn (2)–(4) are derived assuming an inviscid droplet with charge located at the surface. In support of the latter assumption, charged species within a droplet are expected to be either excluded to the droplet surface (hexadecane)^77,78^ or to reorganize the structure of water to localize charge at the droplet surface (aqueous droplets).^19,79^ However, the droplets studied here have measurable viscosities, making the validity of the “inviscid droplet” assumption less obvious. The viscosities of each of the droplet compositions are listed in Table 1. To relate the relative importance of viscous forces to surface tension forces, the Ohnesorge number 
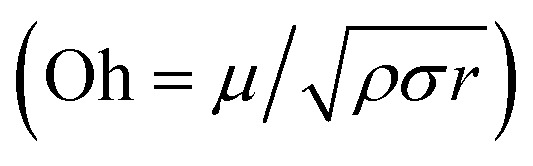
 was calculated for each droplet (Table 1). For the droplets studied here, Oh values are all much less than unity, implying the viscous forces have little effect on the observed oscillation frequency, and the inviscid droplet assumption is valid for the droplet compositions and sizes studied here. Ultimately, the assumptions made to derive eqn (2)–(4) are well supported in the droplets studied here. As a result, despite the large differences in droplet compositions, the shifts in oscillation frequency observed with changing droplet size and charge are well described using the theory first proposed by Rayleigh.

### 3.2 Surface tensions of highly-charged one- or two-component microdroplets

**Fig. 3:**
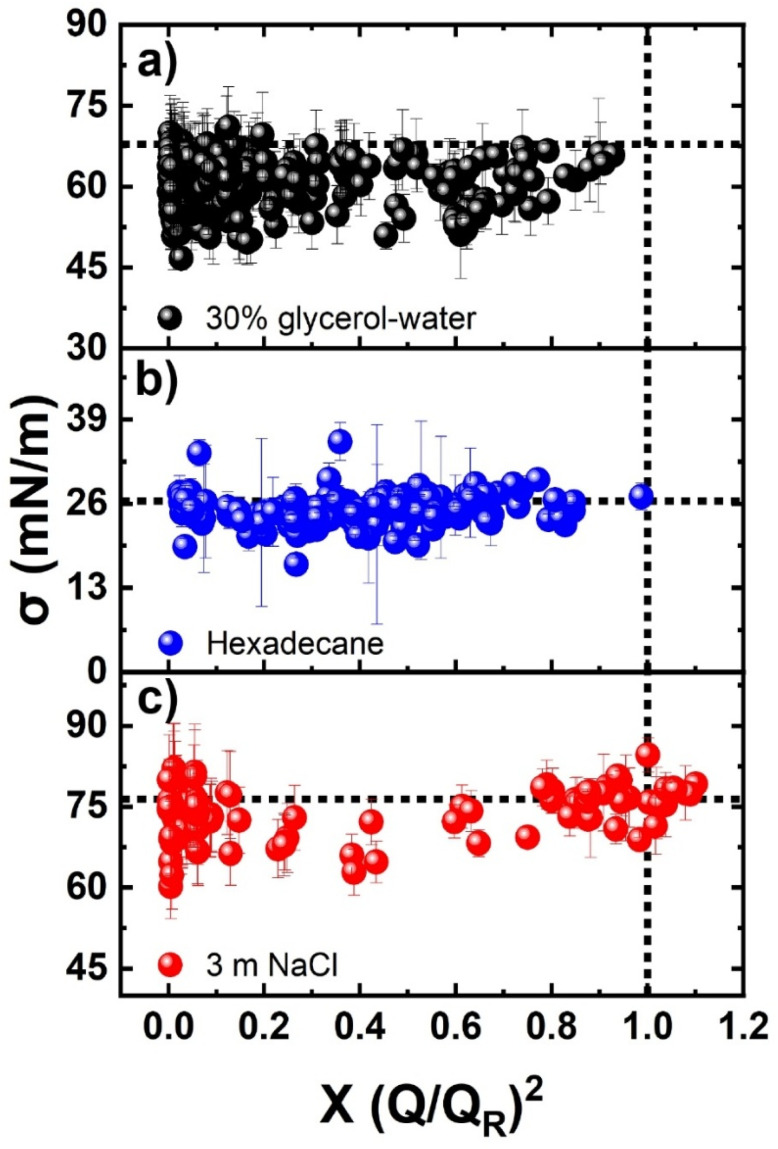
The measured surface tension of 30% glycerol–water (a), hexadecane (b), and 3 m NaCl (c) microdroplets as a function of droplet fissility (*X*). The horizontal black dotted line represents the bulk surface tension of the corresponding droplet system, and the vertical dotted black line indicates the Rayleigh limit.

Having demonstrated observed *ω*_C,rel_^2^ scales appropriately with fissility parameter *X*, we next sought to determine if we could accurately determine the surface tension of highly charged microdroplets from measurements of droplet size, surface oscillation frequency and charge. Fig. 3 shows the experimentally measured surface tension (calculated using eqn (2)–(4)) as a function of fissility parameter *X* for droplets of different compositions. Across all three droplet compositions, the surface tension remained constant as fissility parameter *X* approached 1 and the droplets approached their respective Rayleigh limits. The average surface tensions measured across all values of fissility *X* for the 30% (w/w) glycerol–water, hexadecane, and 3 m NaCl microdroplets were 61 ± 4, 25 ± 3, and 74 ± 5 mN m^−1^, respectively. These average microdroplet surface tensions are in good agreement with the macroscopic surface tension of the different liquids (Table 1 and shown as dotted lines in Fig. 3). Thus, for the droplets measured here, surface tension remains constant regardless of the droplet size and charge. This observation supports the continued use of Rayleigh formalism to predict the stability limit of charged droplets with simple compositions.

Measuring the surface tension of levitated microdroplets requires trapping droplets for upwards of 10 minutes. Thus, while some droplets were observed to undergo coulombic fission in the process of water (or ethanol) evaporation, the droplets used in QELS measurements were inherently stable. As a result, droplets with the highest fissility parameter represent a lower bound of the observed Rayleigh limit. For glycerol–water, hexadecane, and 3 m NaCl microdroplets, the largest fissility parameters measured were 0.93 ± 0.03 0.99 ± 0.03, and 1.10 ± 0.03, respectively. These values of fissility yield observed Rayleigh limits *Q*_R_ that closely match what is predicted by eqn (1) (Table 1). While *Q*_R_ has not been explicitly measured for glycerol–water or NaCl solution microdroplets, experimental values of *Q*_R_ for hexadecane from previous measurements have ranged from 0.73 to 0.98*Q*_R_.^43,46,85^ It is not immediately clear why a larger observed Rayleigh limit is observed in this work compared to previous studies, but it could be the result of measuring droplet size and charge using two separate techniques (which was not done in previous studies). Ultimately, the QELS and charge measurements reported here provide an accurate lower bound of when droplets become unstable with increasing charge. For the droplets studied here, this lower bound limit of electrostatic droplet stability is again well described by the Rayleigh limit (eqn (1)).

### 3.3 Surface tensions of highly charged surfactant-laden microdroplets

**Fig. 4:**
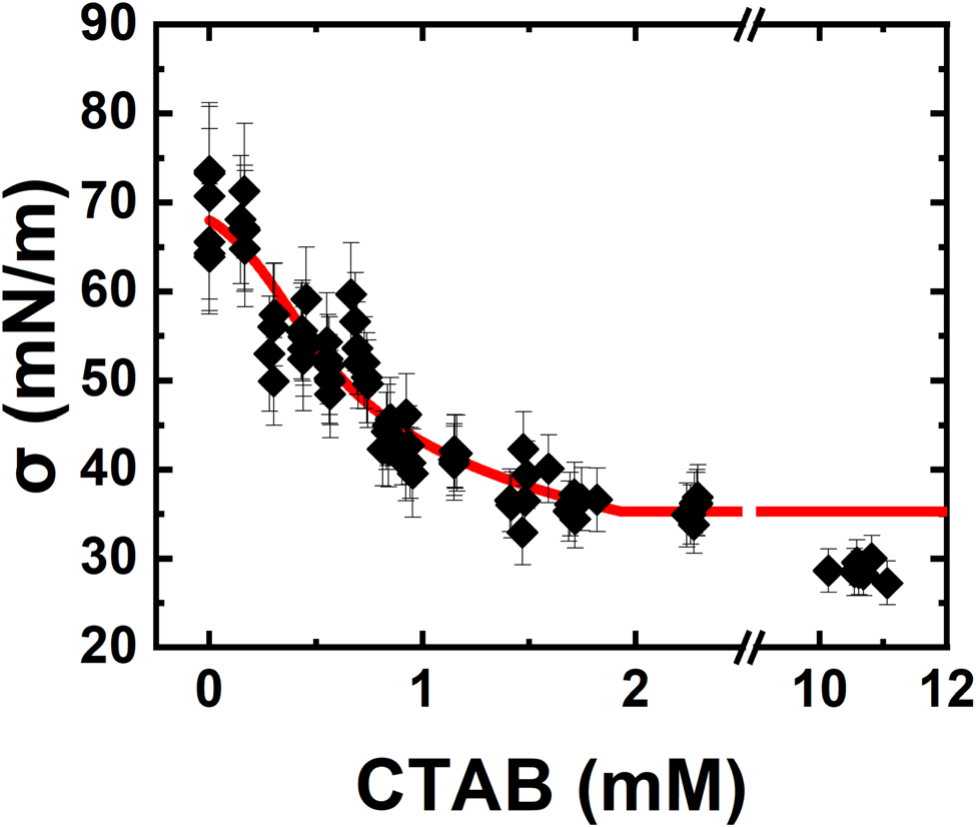
Surface tensions of 30% glycerol–water microdroplets with different total concentrations of CTAB. The data and partitioning model predictions (red line) are reproduced from ref. 50.

It has been predicted (but not experimentally measured) that charges at the surface of a droplet can reorganize the spatial distribution of charged molecules within a droplet, which can influence the composition of the air–water interface.^22,77^ To demonstrate how our new technique to measure surface tensions of highly charged microdroplets can be used to measure charge-induced changes in microdroplet surface composition, we measured the surface tensions of charged droplets containing CTAB surfactant (a cationic surfactant). Fig. 4 shows the surface tension of 8.6 ± 0.4 µm-radius, 30% glycerol–water microdroplets with low fissilities (*X* < 0.1) containing different total concentrations of CTAB. As has been described previously,^57,60^ a slightly larger concentration of CTAB is required to saturate the air–water interface in microdroplets than in macroscale solution. The critical micelle concentration (CMC) in 8.6 µm-radius droplets is ∼1.8 mM compared to 1.2 mM in macroscale solution. Using a simple partitioning model with the Frumkin isotherm, the surface tension in microdroplets can be accurately predicted as a function of total CTAB concentration, providing a baseline for expected behavior in relatively uncharged microdroplets (details in SI).^57^

**Fig. 5:**
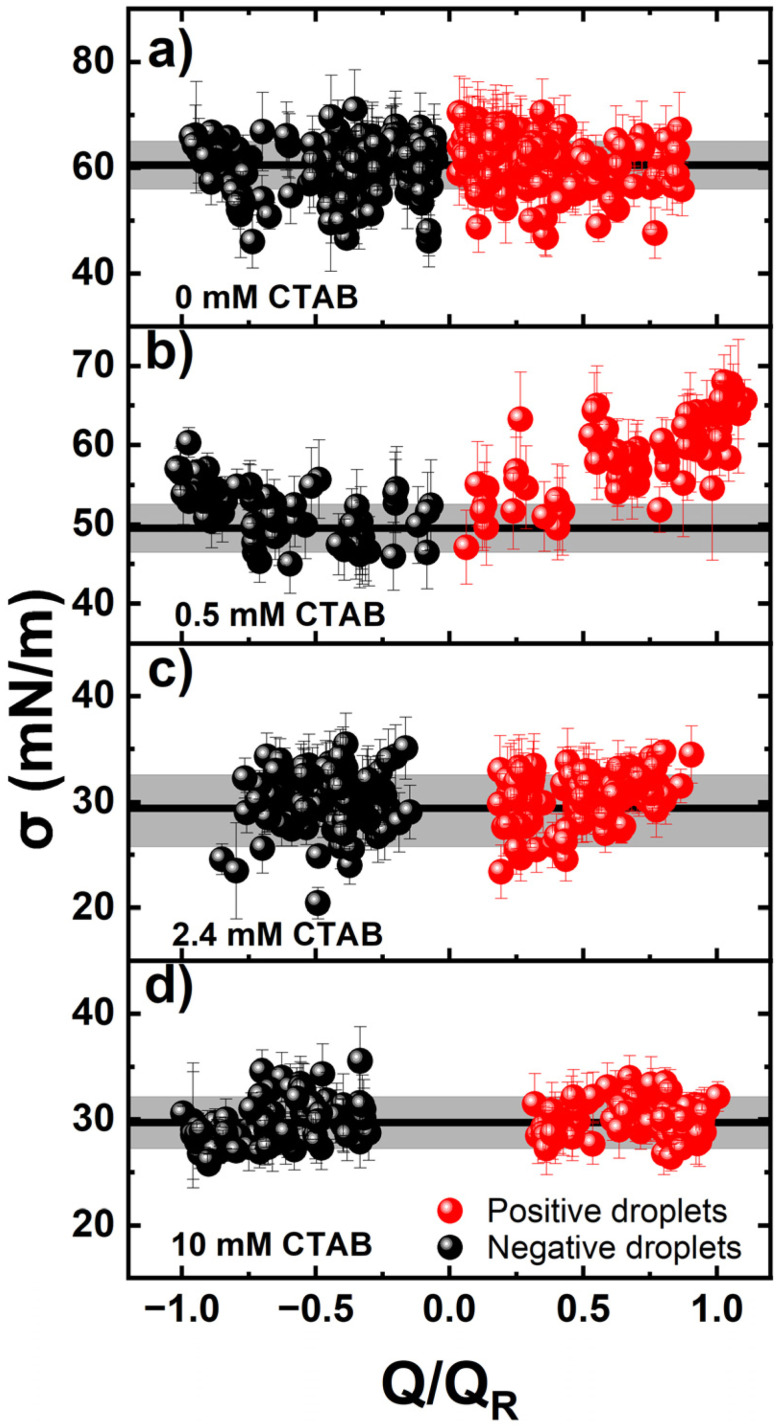
Surface tensions of 30% glycerol–water microdroplets with different net droplet charges and constant total CTAB concentrations of (a) 0 mM, (b) 0.5 mM, (c) 2.4 mM and (d) 10 mM. The solid black lines and shaded regions represent the average surface tension of low fissility microdroplets and the uncertainty in the average (±1 standard deviation).

To explore how net charge on droplets influences the partitioning of charged molecules to the air–water interface, surface tension was measured as a function of charge at three different CTAB concentrations: 0, 0.5, 2.4, and 10 mM. As described earlier, the measured surface tension for microdroplets containing 0 mM CTAB (*i.e.*, 30% glycerol–water microdroplets) remains unchanged with increasing positive or negative charges (Fig. 5a). By comparison, the measured surface tension of 8.1 ± 0.4 µm-radius microdroplets containing 0.5 mM CTAB increased as the magnitude of net charge on the microdroplets increased (Fig. 5b). The surface tension increased from ∼50 mN m^−1^ in low fissility droplets (*X* < 0.1) to ∼65 mN m^−1^ in positively-charged or ∼55 mN m^−1^ in negatively-charged droplets with high fissilities. Experimental surface tension values were not correlated with a change in droplet size or experimental RH (Fig. S2). This suggests that the observed change in droplet surface tension is indeed a consequence of changing droplet charge, and charge can change the partitioning behavior of molecules to the air–water interface.

For 12 ± 2- and 13 ± 2 µm-radius microdroplets containing 2.4 and 10 mM CTAB, respectively, surface tension remains unchanged with increasing net charge on the microdroplet (Fig. 5c and d). At these larger concentrations, there are both sufficiently large ionic strengths to effectively screen interfacial electric fields and sufficient excess CTAB molecules in solution to saturate the air–water interface despite any charge-driven changes in the partitioning behavior CTAB.

For 8.1 µm-radius microdroplets containing 0.5 mM CTAB, the change in surface tension with increasing positive net charge is much more significant than that with negative charge. Again, the difference in behavior with changing charge polarity suggests a charge-driven change in surface composition. While it is possible large electric fields could re-organize the surfactants at the surface to change observed surface tension with increasing charge,^86^ it is expected this effect would be apparent at all concentrations of CTAB and not just those less than the CMC. Thus, the Frumkin isotherm and equation of state were used to calculate the fractional coverage of CTAB for each measured surface tension (Fig. S3). A surface tension of 50 mN m^−1^ (*i.e.*, that of low fissility microdroplets containing 0.5 mM CTAB) corresponds to a CTAB fractional coverage (*θ*_CTAB_) of 0.77, meaning the surface is close to being saturated with CTAB molecules. By comparison, a surface tension of 65 mN m^−1^ (*i.e.*, that of high fissility microdroplets containing 0.5 mM CTAB) corresponds to *θ*_CTAB_ = 0.25 (mostly empty surface). This suggests that most CTAB molecules are stripped from the surface with the addition of net positive charges to the surface of the microdroplet. Using the experimental maximum surface excess concentration of CTAB (1.7 × 10^−6^ mol m^−2^), the volumetric total site concentration in an 8.1 µm-radius microdroplet is ∼0.6 mM.^57^ This represents the maximum concentration CTAB of molecules that can be removed from the microdroplet bulk from surface partitioning. Thus, the transition from *θ*_CTAB_ = 0.77 (low fissility droplets) to *θ*_CTAB_ = 0.25 (high fissility droplets) represents a change in partitioning regime from ‘most CTAB molecules at the microdroplet surface’ to ‘most CTAB molecules in the microdroplet bulk’. An apparent Frumkin adsorption constant (*K*_F_) can be calculated for each of the measured surface tensions using the equilibrium partitioning model.^57^ Assuming all molecules stripped from the surface are dispersed in bulk solution, values of *K*_F_ decrease from ∼1.4 × 10^5^ M^−1^ (macroscale value) to ∼1–3 × 10^3^ M^−1^ in low and high fissility droplets, respectively (Fig. S3). This ∼100-fold change in *K*_F_ is much larger than what would be predicted in macroscale solution for a similarly sized decrease in *θ*_CTAB_ and is a direct consequence of bulk surfactant depletion in microdroplets.^57,60^ Due to their large surface-area-to-volume ratios, a decrease in surface coverage in microdroplets results in a concomitant increase in bulk concentration. Thus, even small changes in microdroplet surface tension can indicate large changes in surfactant partitioning behavior. Here, it is observed that CTAB is a considerably weaker surfactant in highly positively charged droplets than what is observed in macroscale solution.

Using the average droplet size (*r*_drop_ = 8.1 ± 0.4 µm) and experimental maximum surface excess concentration of CTAB, roughly 4.3 × 10^8^ CTAB molecules are removed from the surface as droplet charge increases from low fissility *Q* to high fissility *Q*. By comparison, the maximum positive charge measured on a droplet was 458 ± 1 fC, which corresponds to 2.9 × 10^6^ charge carriers. At this largest measured charge, it is calculated that each extra positive charge carrier displaces roughly 290 CTAB molecules from the surface. Thus, there exists a large discrepancy between the number of CTAB molecules displaced from the surface and the number of charge carriers in the droplet.

It is useful to consider the electrostatic potential of the droplet surface to rationalize how such a small number of charges at the droplet surface can influence molecular partitioning. The surface tension measurements in this work are made on second timescales and thus report on the average surface potential at the air–water interface. They are not expected to be sensitive to large, localized fluctuations of the electric field at the air–water interface.^87^ The net charge on droplets in electrodynamic levitation experiments is typically ascribed to an imbalance of ions within droplets.^88,89^ It is assumed that extra CTA^+^ cations are not the charge carriers in the positively charged droplets because the number of charge carriers driving changes in droplet surface tension is much smaller than the number of CTAB molecules displaced. Instead, charge carriers are likely excess protons or hydroxide ions facilitated by the preferential adsorption of hydronium to the silica capillary in the droplet dispenser.^90^ As charge is added to the droplet surface, the electric potential at the surface increases according to 
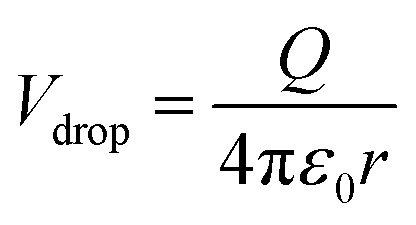
. For example, the surface potential of the microdroplet with the highest measured positive charge is ∼490 V. This large electrostatic potential is screened in the droplet interior, according to the Debye screening length *λ*_D_:6
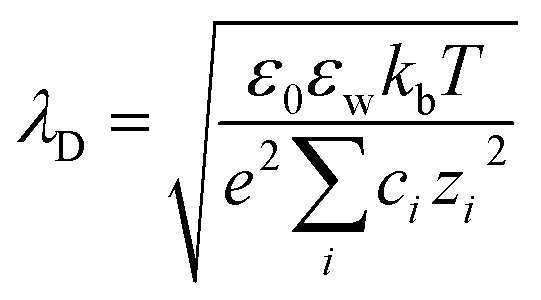
where *ε*_w_ is the dielectric constant of water, *k*_b_ is Boltzmann's constant, *T* is absolute temperature, *e* is the elementary charge, and *c*_*i*_ and *z*_*i*_ are the number densities and valencies of the electrolyte ions. The Debye screening length of a 0.5 mM CTAB solution is 13.6 nm, meaning the large electrostatic potential is shielded completely within 10 s of nm from the droplet surface. However, *λ*_D_ is significantly longer than the length of a CTAB molecule (∼2 nm).^91^ Thus, as charge on droplets increases, positively charged CTA^+^ ions experience strong electrostatic repulsion from large positive electrostatic potential at the air–water interface over a length scale longer than molecule. This leads to their exclusion from the air–water interface, as was previously predicted.^22^ It is likely that the ability of large surface potentials to influence molecule partitioning is highly dependent on ionic strength. For example, as CTAB concentration increases and *λ*_D_ approaches the length scale of a single molecule (such as the case for 2.4 and 10 mM CTAB), the electric potential from the net charge may be screened effectively and not result in changes in CTAB partitioning behavior.

Using similar rationalizations, it is expected that the surface tension of CTAB-laden microdroplets would decrease in negatively charged droplets (*i.e.*, the large negative electrostatic potential would promote partitioning of CTA^+^ to the air–water interface). Instead, it is observed that the surface tension increases slightly from ∼50 mN m^−1^ in low fissility droplets to ∼55 mN m^−1^ in high fissility droplets. If all CTAB molecules in the microdroplet partitioned to the surface, the maximum *θ*_CTAB_ for an 8.1 µm-radius microdroplet containing 0.5 mM CTAB is 0.79. From the Frumkin equation of state, this corresponds to a surface tension of ∼48 mN m^−1^, which is only slightly smaller than the surface tension of uncharged droplets. Because of bulk depletion effects, a large decrease in surface tension is not possible for surfactant systems where most of the molecules already reside at the air–water interface. Thus, the small observed increase in droplet surface tension with larger net negative charge could instead arise from a small systematic error in droplet size/charge measurements or the bulk depletion of CTAB molecules in microdroplets from electrostatic repulsion during droplet formation. Additionally, the small increase in droplet surface tension could arise from electric field-induced reorientation of interfacial CTAB molecules.^86^

Charge-driven changes in surface tension complicates our understanding of the stability criteria of microdroplets containing charged surfactants. A fissility parameter >1 indicates droplets contain more charge than *Q*_R_. The lower bounds of the maximum fissility parameters for positively and negatively charged microdroplets containing 0.5 mM CTAB are 1.24 ± 0.05 and 0.96 ± 0.07, respectively. This indicates positively charged droplets can carry ∼11% more charge than is predicted using the low fissility surface tension, while negatively charged droplets still fragment at the theoretical charge. While values of *X* > 1 have been observed recently for aqueous nanodroplets undergoing evaporation,^92^ most experimental determinations of the Rayleigh limit in levitated microdroplets observe the Rayleigh limit to be at or slightly below the predicted value.^43,45,46,49^ For example, microdroplets containing 1 wt% sodium dodecyl sulfate (an anionic surfactant) underwent droplet fission at 90% of the theoretical Rayleigh limit.^45^ Importantly, the concentration at 1 wt% is significantly larger than the CMC of sodium dodecyl sulfate. Similarly, when CTAB concentrations increase, the difference in behavior between positively and negatively charged droplets disappears and droplets fission close to the Rayleigh limit. For microdroplets containing 10 mM CTAB, the maximum fissility parameters in positively and negatively charged microdroplets are 1.01 ± 0.03 and 1.03 ± 0.02, respectively. Thus, these results again highlight that charge-driven changes in microdroplet surface tension can result in stable droplets containing more charge than is predicted by the Rayleigh limit. However, this effect is likely limited to surfactant concentrations smaller than the microdroplet CMC.

Ultimately, with the new ability to measure the surface tension of highly charged microdroplets, we have demonstrated that net charge can influence equilibrium partitioning of surfactants to the air–water interface in microdroplets. This could have implications for understanding accelerated chemistry in microdroplets as most observations of accelerated chemistry have used highly charged droplets.^93^ Accurate representations of the concentrations of molecules at the surface of microdroplets is crucial to understanding and accurately modeling chemistry in microdroplets.^6,94^ For example, while the strong surfactants used in this work highlight how large surface charge densities can deplete charged molecules from the air–water interface, it is expected that charged molecules could also be promoted to the air–water interface. For less surface-active molecules or charged intermediates, this electrostatic driven enrichment could increase local concentrations to promote chemistry in microdroplets.^22^ This effect is likely to be stronger in smaller charged droplets (*e.g.*, nanodroplets) with higher curvature and stronger surface electric fields, but future studies are required to understand how charge-driven changes in surface partitioning scales with droplet size and ionic strength. Ultimately, the new technique described here will facilitate future studies exploring charge-driven changes in the concentrations of molecules at the air–water interface of microdroplets.

## 4. Conclusion

In conclusion, we have presented a new methodology to measure the surface tensions of highly charged microdroplets levitated within a QET. Using simple single- or double component droplets with distinct chemical compositions, we observed that the surface oscillation frequency of levitated microdroplets predictably decreases with increasing net charge. By accurately quantifying droplet surface oscillation frequency, size, and charge, we demonstrated that formalism described by Rayleigh accurately predicts the effect of charge on the resonant surface oscillation frequencies of levitated droplets. As a result, using Rayleigh's predictions, we demonstrated that surface tensions of droplets can be measured for droplets with net charges that approach the Rayleigh limit.

Droplet charge is thought to play an important role in promoting reactivity in microdroplets by influencing reaction thermodynamics^1,2^ or altering distribution of molecules within droplets.^22^ The latter could influence both the stability of charged microdroplets (*e.g.*, by changing surface tension to change *Q*_R_) and affect microdroplet reactivity (*e.g.*, by altering surface concentrations of charged reactants). To demonstrate this, we used our new technique to measure the surface tensions of highly charged droplets containing CTAB (a cationic surfactant). For CTAB concentrations less than the microdroplet CMC, the surface tension of microdroplets was observed to increase with increasing positive charge, suggesting the positive charges at the surface decrease the propensity of CTAB for the surface. A similarly sized increase in surface tension was not observed in negatively charged droplets nor droplets with CTAB concentrations larger than the CMC. These initial experiments demonstrate that there are some conditions where droplet charge can alter molecular partitioning to the air–water interface and influence droplet stability criteria. Ultimately, measuring the surface tensions of highly charged microdroplets using the methodology described in this work will directly inform how charge can alter interfacial concentrations of molecules to promote microdroplet chemistry. This will serve to advance our understanding of microdroplet chemistry in atmospheric, biological and industrial contexts.

## Author contributions

M. I. J. conceptualized the project, M. I. J. and F. A. performed the experiments, analyzed and interpreted the experimental data, and wrote the manuscript.

## Conflicts of interest

There are no conflicts to declare.

## Supplementary Material

SC-017-D6SC03152H-s001

## Data Availability

Data are available from the corresponding author upon request. In addition, all data underlying the figures are available through the Texas Data Repository at https://doi.org/10.18738/T8/O1UWWP. Supplementary information (SI): description of modeling surface tension of CTAB-laden microdroplets; Frumkin adsorption parameters for CTAB; normalized frequency shifts for positively and negatively charged glycerol–water microdroplets; correlation plots between surface tension and radius/RH for charged microdroplets containing 0.5 mM CTAB; calculated surface coverages and apparent *K*_F_ values of CTAB for differently charged 0.5 mM CTAB-laden microdroplets. See DOI: https://doi.org/10.1039/d6sc03152h.
