# Supplementary material for: Surface tension measurements reveal charge-driven surfactant depletion in microdroplets approaching the Rayleigh limit
Source: Chem Sci. 2026 Jun 11;17(29):14194–203. doi: 10.1039/d6sc03152h (PMC13271853; doi:10.1039/d6sc03152h)
Supplement: SC-017-D6SC03152H-s001 [file SC-017-D6SC03152H-s001.pdf]

Supplementary Information (SI) for Chemical Science.
This journal is © The Royal Society of Chemistry 2026

Supplementary Information for:

Surface Tension Measurements Reveal Charge-Driven Surfactant Depletion in Microdroplets Approaching the Rayleigh Limit

Firoz Ahmed[1] and Michael I. Jacobs[1*]

[1]Department of Chemistry and Biochemistry, Texas State University, San Marcos, TX 78666

*Corresponding author: mijacobs@txstate.edu

Contents:

Page S-2 Description of modeling surface tension of CTAB-laden microdroplets.
Table S1. Frumkin adsorption parameters for CTAB.
Page S-3 **Figure S1.** $\omega_{C,rel}^2$ vs. fissility for positively and negatively charged droplets.
Page S-4 **Figure S2.** Correlation plots between surface tension and radius/RH for charged microdroplets containing 0.5 mM CTAB.
Page S-5 **Figure S3.** Calculated surface coverages and K_F values of CTAB for differently charged 0.5 mM CTAB-laden microdroplets.
Page S-6 Supporting Information References

Modeling surface tension of CTAB-laden microdroplets.

The Frumkin isotherm[1,2] is used to relate the fractional surface coverage of CTAB (θ_{CTAB}) at the air-water interface to the bulk concentration of CTAB according to:

$$K_F[CTAB]_{bulk} = \frac{\theta_{CTAB}}{1-\theta_{CTAB}}\exp(-A\theta_{CTAB}) \tag{S1}$$

where K_F is the Frumkin equilibrium constant and A is a constant that accounts for the interactions between adsorbed CTAB molecules (positive values of A indicate cooperative interactions, negative values of A indicate anti-cooperative interactions). The corresponding Frumkin equation of state that relates $[CTAB]_{bulk}$ to solution surface tension σ is:

$$\sigma = \sigma_0 + n\,\Gamma_\infty RT\left[\ln(1-\theta_{CTAB}) + \frac{A}{2}(\theta_{CTAB})^2\right] \tag{S2}$$

where σ_0 is the surface tension of solution without surfactant, n is the constant that depends on how many molecules adsorb to the interface (n = 2 for CTAB), Γ_∞ is the monolayer surfactant concentration, R is the gas constant and T is absolute temperature. The previously determined best fit parameters for CTAB adsorption to the air-water interface in 30% (w/w) glycerol water are provided in Table S1.[3] A custom python script was used to explore how θ_{CTAB} changes with measured surface tension using the Eq. S2.

An equilibrium surface partitioning model is used to describe the surface tension of microdroplets containing CTAB molecules. This partitioning model was previously described in detail.[3] Briefly, the volumetric total site concentration $[site]_{total}$ is determined from the droplet radius and Γ_∞ according to $[site]_{total} = 3\Gamma_\infty/r$. For a given microdroplet size, $[site]_{total}$ is conserved such that

$$[\text{site}]_{\text{total}} = [\text{CTAB}]_{\text{ads}} + [\text{site}], \tag{S3}$$

where $[CTAB]_{total}$ and [site] are the volumetric concentrations of adsorbed CTAB and empty sites, respectively. Furthermore, because there are a fixed number of CTAB molecules in the droplet, the total concentration of CTAB is conserved:

$$[CTAB]_{total} = [CTAB]_{ads} + [CTAB]_{bulk}. \tag{S4}$$

For a given $[CTAB]_{total}$ and droplet size, a custom python script is used to solve for θ_{CTAB} using the Frumkin isotherm (Eq. S1) and Eqs. S3 and S4. The surface tension of the droplet is then calculated using the Frumkin equation of state (Eq. S2). The minimum surface tension from macroscale measurements (35 mN/m) is used when the predicted surface tension is less than the surface tension at the critical micelle concentration (CMC).

Table S1. Frumkin adsorption parameters for CTAB in 30% (w/w) glycerol-water.

Γ_∞ ×10^{-6} (mol m^{-2})	K_F (M^{-1})	A	$[CTAB]_{CMC}$ (mM)
1.7±0.2	(1.4±0.6) ×10^5	-2.6±0.4	1.2

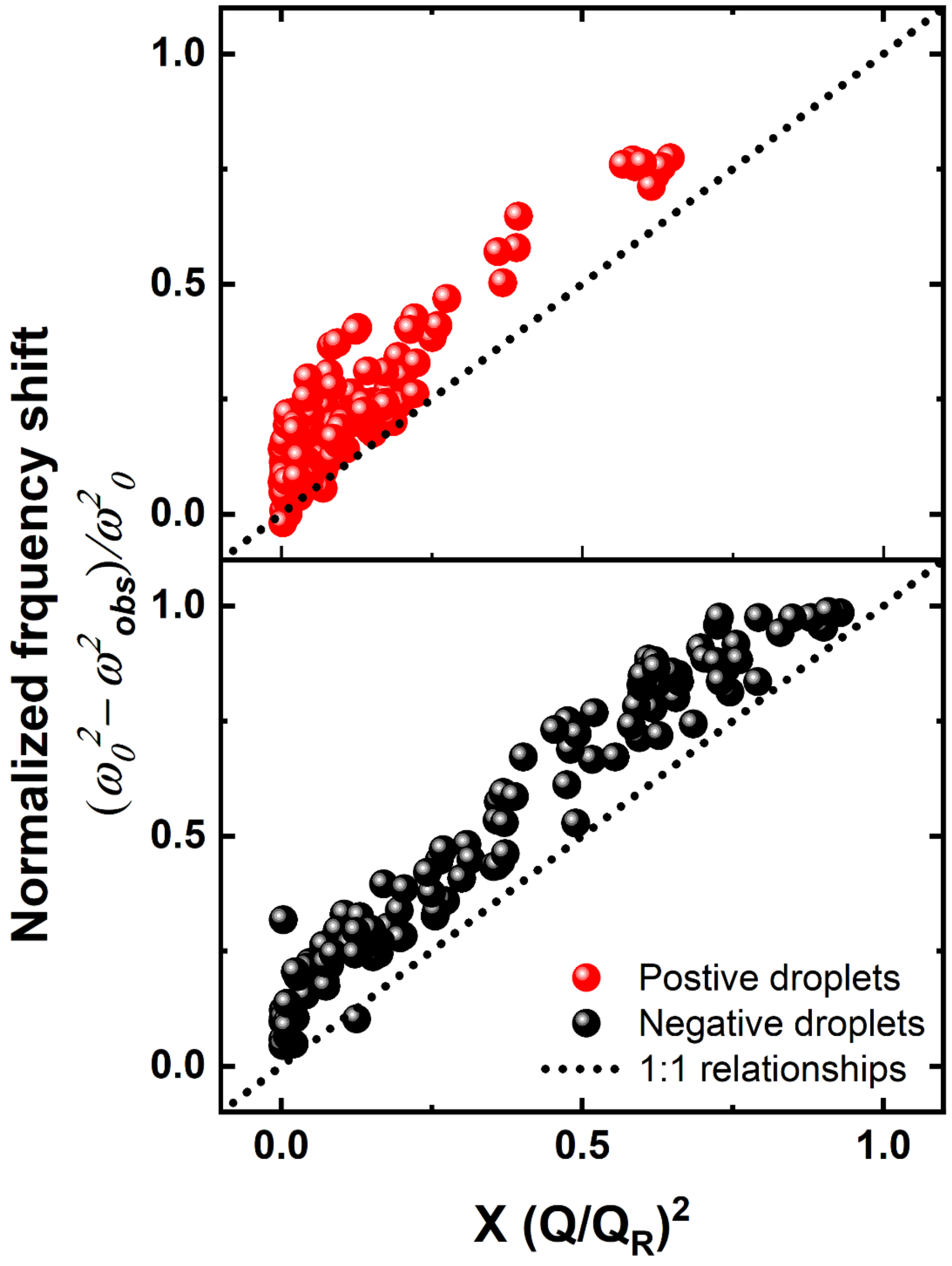

Figure S1. Relationship between the normalized frequency shift and fissility for positively and negatively charged of 30% (v/v) glycerol-water microdroplets.

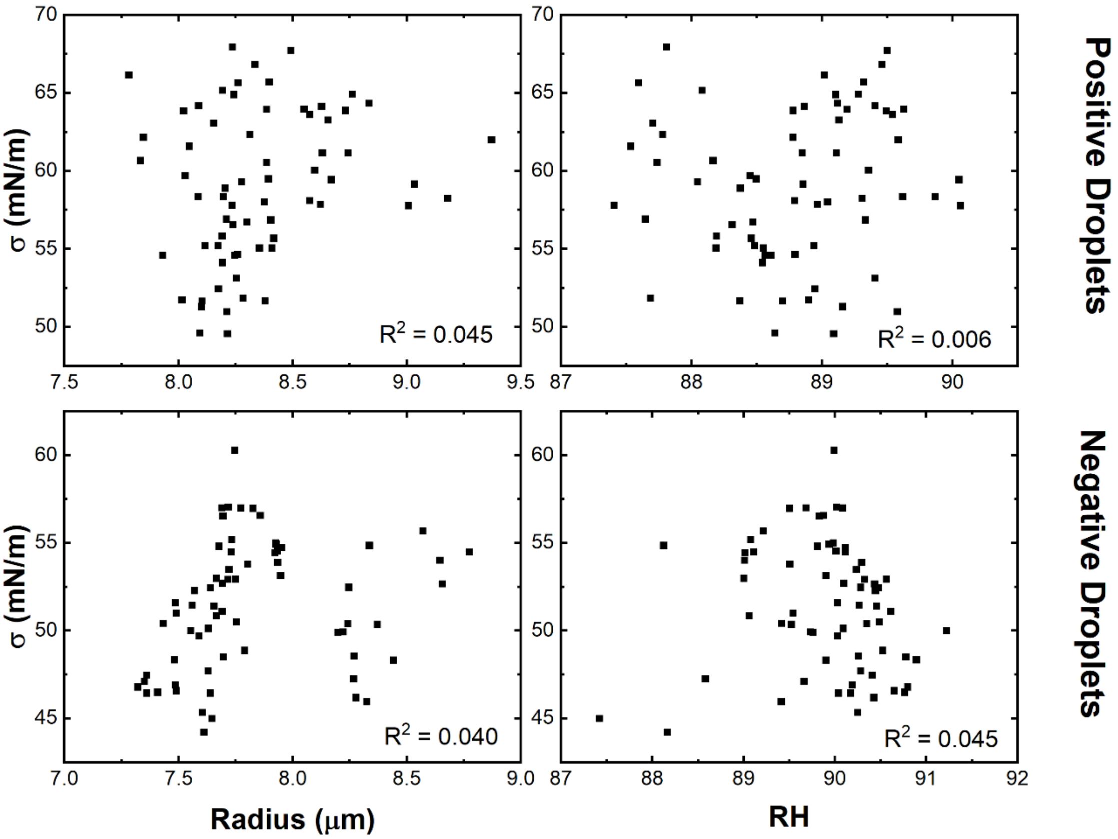

Figure S2. Correlation plots between surface tension and droplet size/relative humidity (RH) for positively and negatively charged 30% (v/v) glycerol–water microdroplets containing 0.5 mM total CTAB.

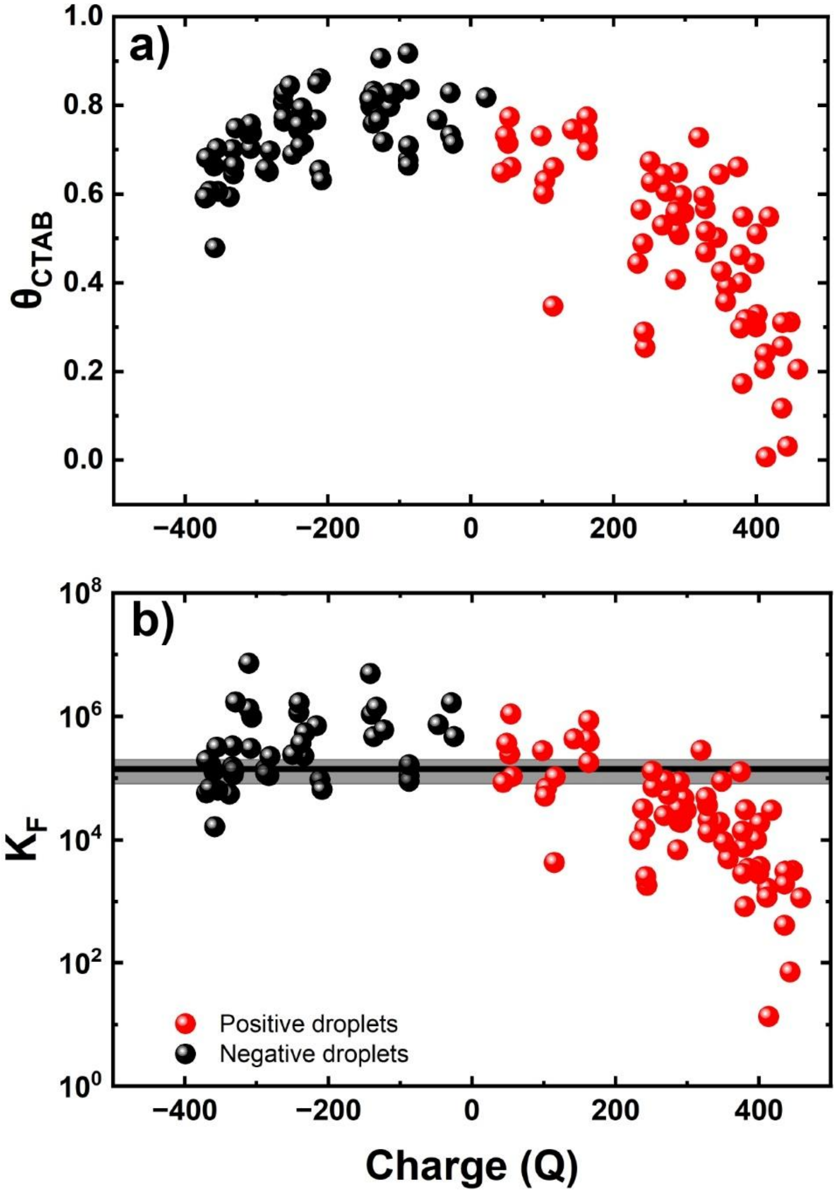

Figure S3. a) Surface coverage of CTAB (θ_{CTAB}) vs. net droplet charge in 8.1-μm-radius microdroplets containing 0.5 mM CTAB calculated using surface tension measurements and the Frumkin isotherm. b) Apparent Frumkin adsorption coefficients (K_F) of CTAB vs. net droplet charge in 8.1-μm-radius microdroplets containing 0.5 mM CTAB. The solid horizontal line represents K_F of CTAB in 30% glycerol-water.

References

1 V. L. Kolev, K. D. Danov, P. A. Kralchevsky, G. Broze and A. Mehreteab, *Langmuir*, 2002, **18**, 9106–9109.

2 C. T. Hsu, C. H. Chang and S. Y. Lin, *Langmuir*, 1997, **13**, 6204–6206.

3 M. I. Jacobs, M. N. Johnston and S. Mahmud, *Journal of Physical Chemistry A*, 2024, **128**, 9986–9997.
